# Assessment of Knowledge, Attitude, and Practice Towards the Prevention of Monkeypox Among People of Bangladesh With at Least Primary Education: A Cross‐Sectional Study

**DOI:** 10.1002/hsr2.71560

**Published:** 2025-11-26

**Authors:** Radwan Raquib, Proloy Chakraborty Tusher, Anisa Ahmed Chowdhury, Obaidul Islam, Jahid Hasan Tipu, Himel Talukder, Mohammod Misbah Uddin, Ahsan Raquib, Tilak Chandra Nath

**Affiliations:** ^1^ Department of Parasitology Sylhet Agricultural University Sylhet Bangladesh; ^2^ Department of Statistics Shahjalal University of Science and Technology Sylhet Bangladesh; ^3^ Laboratory of Veterinary Epidemiology, College of Veterinary Medicine Chungbuk National University Chungbuk Republic of Korea; ^4^ Department of Clinical Science, Faculty of Medicine University of Bergen Bergen Norway; ^5^ Department of Geography and Environmental Sustainability University of Oklahoma Norman Oklahoma USA; ^6^ Faculty of Veterinary Medicine University of Calgary Calgary Alberta Canada; ^7^ Department of Health Management, Atlantic Veterinary College University of Prince Edward Island Charlottetown Prince Edward Island Canada

**Keywords:** Bangladesh, KAP, monkeypox, prevention and control, public health

## Abstract

**Background and Aims:**

The recent emergence and rapid spread of monkeypox (Mpox) have become a global public health concern. Gaps in proper information dissemination can cause confusion and knowledge divergence among the general public, leading to difficulties in disease management. Therefore, this study aimed to assess the overall knowledge, attitude, and practice (KAP) regarding Mpox among the general population of Bangladesh to help implement optimal public health interventions.

**Methods:**

A cross‐sectional study was conducted using an online questionnaire survey to evaluate the KAP related to Mpox among the general population of Bangladesh. A total of 1025 KAP domains were registered during the survey, among which 938 were eligible for final analysis. Comparisons between different groups were performed using the Wilcoxon signed‐rank and the Kruskal–Wallis test. Additionally, a multivariable logistic regression analysis was conducted to identify factors associated with williness to practice preventive behaviours against Mpox.

**Results:**

Only 7.25% (68/938) of participants demonstrated good knowledge, 20.58% (193/938) had positive attitudes, and 25.05% (235/938) were willing to practice preventive measures toward Mpox prevention, indicating overall inadequate KAP levels. The Kruskal–Wallis test revealed statistically significant differences in knowledge scores based on education level (*p* value = 0.004), age group (*p* value = 0.008), and division (*p* value = 0.009). Similarly, significant variations in attitude scores were observed across areas of residence (*p* value = 0.003), education level (*p* value < 0.001), and age group (*p* value = 0.002). Furthermore, both univariate and multivariable logistic regression analyses identified gender, knowledge level, and attitude score as significant predictors of willingness to engage in preventive practices.

**Conclusion:**

The low KAP scores suggest an urgent need for mass awareness campaigns for Mpox prevention in Bangladesh. The findings of this study could assist policymakers in taking necessary steps to mitigate the potential public health burden associated with Mpox in the country.

## 1 Introduction

Monkeypox (Mpox) is a neglected emerging zoonotic pathogen caused by the monkeypox virus, a double‐stranded DNA virus belonging to the genus Orthopoxvirus [1, 2, 3]. The first Mpox case was reported in 1958 from monkeys kept in captivity in Denmark, and the first human case of Mpox was reported in 1970 in the Democratic Republic of Congo [4]. The disease remained endemic in the rainforests of central and western Africa until 2003 [5], when an Mpox case was reported in the USA in patients exposed to prairie dogs, which was the first detected case of Mpox outside Africa, and then it steadily spread around the globe [5, 6]. Though it is less severe with a low case fatality rate, coinfection, especially with SARS‐COV‐2, can increase both pathogenicity and infectivity [7].

Human Mpox virus has two clades: clade I, which is endemic in Central Africa and is more severe, and clade II, which originates from West Africa [3]. Since January 2022, there were 102,000 recorded cases of Mpox (clade II) worldwide, with 220 deaths [8]. In 2024 alone, about 9000 Mpox (clade II) cases were reported globally [8] and the World Health Organization (WHO) declared Mpox a Public Health Emergency of International Concern [9]. Although Mpox has not yet been reported in Bangladesh, it has already been reported in the neighboring country, India [10]. Due to close geographical proximity to India and the frequent movements of humans and animals across the border, there is a potential risk of the emergence and spread of Mpox in Bangladesh in the near future.

Clinical symptoms of Mpox resemble those of smallpox and include painful skin lesions (typical pox lesions like macules, papules, vesicles, pustules, etc.), fever, headache, myalgias, and malaise [11, 12, 13]. Although this virus has similarities with smallpox in symptoms, it shows additional lymphadenopathic symptoms [4]. Transmission of Mpox occurs by direct contact with skin lesions, bodily fluids, fomites, and large respiratory droplets [12]. Although it is not a typical sexually transmitted disease, intimate physical contact, particularly among homosexual men, increases the risk of Mpox transmission [14].

From a disease prevention perspective, it is imperative to consider the public viewpoint on the disease, their knowledge and attitudes towards it, and their willingness to contribute to the prevention of the disease. Furthermore, for a lower‐middle‐income densely populated country like Bangladesh with low public health infrastructure, the introduction of new zoonotic infectious diseases will be particularly vulnerable, causing mortality and economic challenges. Proactively assessing the general public's knowledge, attitude, and willingness to practice preventive behavior is crucial to identify areas for improvement and help policy‐makers implement strategies [4, 14].

To address this need, this study incorporated participants from almost all regions of Bangladesh to assess the level of knowledge, attitude, and practice (KAP) of Mpox, as well as factors associated with willingness to do preventive practices among individuals in Bangladesh with at least primary education.

## 2 Materials and Methods

### 2.1 Sampling Method and Participants

A cross‐sectional study was conducted between September 2024 and January 2025 using an anonymous online questionnaire (Google form) to evaluate the KAP regarding Mpox among people of Bangladesh with at least primary level of education. The questionnaire was prepared in both Bangla and English. The questionnaire was circulated via emails (through authors' academic contacts across different universities) and multiple social media platforms (Facebook, Messenger, and WhatsApp). A convenient snowball sampling approach was used, whereby initial participants were encouraged to share the link within their networks. Participation was voluntary, and informed consent was obtained at the beginning of the form. Responses were automatically recorded in Google Sheets and later exported to Microsoft Excel for data cleaning, and subsequent statistical analysis.

The inclusion requirements for the participants must be a citizen of Bangladesh, able to read and understand English or Bangla, be above 15 years of age, and have at least a primary level of education.

### 2.2 Bias

Efforts were made to minimize potential sources of bias. To reduce social desirability bias, the questionnaire was completely anonymous. We shared the survey widely through both email and multiple social media platforms to reach participants from diverse backgrounds. Clear inclusion criteria were used to reduce selection bias. In addition, we used standardized questions adapted from previously validated KAP surveys or from published articles to minimize measurement bias.

### 2.3 Study Size

As this was an exploratory study using a convenient snowball sampling approach, no formal sample size calculation was performed; however, the minimum sample size was estimated using the single proportion formula at a 95% confidence level, with *p* = 0.5 (expected proportion), *d* = 0.05 (margin of error), yielding 384 participants. All responses collected during the data collection period (September 2024–January 2025) that met the inclusion criteria were included in the final analysis.

### 2.4 Questionnaire Design

Following several published literature [4, 15, 16] the questionnaire for this study was generated considering socio‐cultural and geographic aspects, which were divided into four sections. The sociodemographic characteristics of the participants included questions related to gender, location (division and district), area (urban/rural), age, education, occupation, and marital status. The knowledge section included 11 questions; among these, six questions allowed responses as “Yes,” “No,” and “Don't know”, two questions were multiple‐choice, and three questions allowed multiple answers (Supporting Information S1: Supplementary Table 1). The attitude section comprised 10 questions, with responses ranging from “Strongly Agree” to “Strongly Disagree” (Supporting Information S1: Supplementary Table 2). To evaluate participants' willingness to practice towards the prevention of Mpox, we designed nine practice‐related questions (Supporting Information S1: Supplementary Table 3). To check the reliability of KAP scores, Cronbach's alpha values were calculated separately for each KAP‐related question.

### 2.5 Statistical Analysis

Initially, the KAP domain of 1025 participants was included. Because this study did not involve in‐person interviews, it was unlikely to receive responses from individuals who were illiterate; therefore, they were excluded. According to the 2022 Population and Housing Census, the literacy rate in Bangladesh is 74.66% [17], meaning the study population represents roughly three‐quarters of the country's people. It is also reasonable to assume that KAP regarding Mpox may be lower among illiterate individuals. Regardless of specific influencing factors, this group would still require targeted awareness and education campaigns. Given Bangladesh's limited resources, it is practical to first identify the factors influencing KAP among the literate population and then prioritize awareness efforts for groups most in need. After omitting missing observations and filtering ineligible participants (aged below 15) due to their small contribution to the study population, 938 KAP responses of participants were taken for final analysis. Data were then coded to transform them into an analyzable form. KAP‐related questions were coded based on right and wrong answers, and for each person, cumulative KAP scores were generated.

Descriptive statistics were performed to calculate the frequencies, percentages, and interquartile ranges. To compare the KAP score across different predictors, the normality of the scores was tested in different categories of predictors using the Shapiro–Wilk Normality test. As all the data were non‐normally distributed, either the Wilcoxon signed‐rank test or the Kruskal–Wallis test was performed based on the number of categories in each predictor.

We employed a multivariable logistic regression approach using willingness to practice as our primary outcome, as implementation of proper practice reduces transmission and mitigates spread. For this, we categorized individual participants' practice scores as “good” if they scored ≥ 75% of the achievable score, otherwise categorized as “bad”. As the knowledge (good or bad knowledge) and attitude (good or bad knowledge) of a person lead to practice behavior, we categorized them similarly as practice scores.

As the structure of our data is hierarchical, with different levels like division, district, and participants, in both univariate and multivariable models, we ran the analysis with and without taking into account the random effects. In the univariate model, we estimated the association between the practice level and all predictors individually, once at a time. A predictor was included in the multivariable model if a *p* value less than 0.20 was obtained in the univariate analysis. The initial multivariable model was run with all the predictors with a *p* value less than 0.20 in the univariate analysis. Knowledge level and attitude level were our two main exposures. During model building, any additional predictors that changed the magnitude of the coefficients for these two exposures by more than 20% were considered as potential confounders and were therefore retained in the model. Although we developed a causal diagram (not shown) and used terms such as “confounding”, it is important to note that this modeling approach was conducted solely for predictive purposes. The results should be interpreted as evidence of causal association.

The interactions among all the significant predictors in the multivariable model were also checked, and there was no significant interaction among the predictors retained in the multivariate logistic regression model. We compared different multivariable models with and without random effects using Akaike Information Criterion (AIC) and Bayesian Information Criterion (BIC) values. In addition, multicollinearity between predictors of the final model was evaluated using the Variance Inflation Factor (VIF). As adding random effects did not add any additional value to the model, we decided to stick to the model without random effects (Table 1).

**Table 1:** Results of comparison between different models, which implies the model without random effect (RE) was better.

Model	AIC	BIC
Model 1 (No RE)	972.57	1001.64
Model 2 (RE: division)	973.71	1007.61
Model 3 (RE: district)	973.71	1007.61
Model 4 (RE: division + district)	974.24	1008.15

Odds ratios of the univariate and multivariable models were reported with a 95% confidence interval. The overall model fit was assessed using the Hosmer–Lemeshow goodness‐of‐fit test, where a nonsignificant *p* value was considered indicative of an adequate fit. Model fit was evaluated using Pearson residuals to assess how well the model predictions aligned with the observed data. In addition, standardized residuals and Cook's distance were examined to identify potential outliers and influential observations. The predictive power of the model was evaluated using the area under the Receiver Operating Characteristic (ROC) curve. All statistical analyses were performed using R Studio (version 4.4.2; packages: ltm, pROC, rstatix, and car) and Stata (version 17). All tests were two‑sided, with *p* values < 0.05 were considered statistically significant.

### 2.6 Ethical Considerations

The study was performed in accordance with the Declaration of Helsinki [18] ensuring respect for participants, their informed consent, ethical oversight, and scientific integrity. This study protocol was reviewed and approved by the Sylhet Agricultural University Research System and the Department of Parasitology, Sylhet Agricultural University, Bangladesh (Approval number: SAURES‐UGC‐2023‐2024‐02). Before sending the Google form containing the questionnaire, participants provided their consent to participate. No personal identifiable information was collected, thereby ensuring participants' confidentiality.

## 3 Result

### 3.1 Demographic Characteristics of the Participants

Of the 938 eligible participants, 54.3% (509/938) were male, and the remaining were female. Most of the participants (551/938, 58.7%) were between 22 and 26 years old, 23.1% (217/938) were less than or equal to 21 years old, and 18.1% (170/938) were older than 26 years. Participants were predominantly well‐educated, with 60.1% (565/938) being undergraduate students and 30.2% (283/938) pursuing or holding a graduate or higher degree. Regarding occupation, 52.6% (493/938) of participants were students, 36.2% (340/938) were employed, and the remaining were unemployed. In terms of residential areas, 71.4% (670/968) were from urban areas, while 28.7% (268/938) were from rural areas. The majority of the participants were from Sylhet (366/938, 39.0%), Dhaka (189/938, 20.2%), and Chattogram (137/938, 14.6%), with the remaining 26.2% (246/938) from the other five divisions of Bangladesh (Table 2).

**Table 2:** Sociodemographic characteristics and median (IQR) scores for knowledge, attitude, and practice among respondents.

Characteristics		Number (%)	Knowledge score				Attitude score				Practice score			
			Median	IQR (Inter‐quartile range)	W/H statistic	*p* value	Median	IQR (Inter‐quartile range)	W/H statistic	*p* value	Median	IQR (Inter‐quartile range)	W/H statistic	*p* value
Gender	Female	429 (45.74)	8	8	116,847	0.060	12	7	108,687	0.905	15	5	126,857	< 0.001
	Male	509 (54.26)	7	8			12	7			14	5		
Area	Rural	268 (28.57)	8	9	84,008	0.120	11	7	78,665	0.003	14	4	86,402	0.366
	Urban	670 (71.43)	7.5	7			12	7			14	5		
Education	Higher secondary or less	91 (9.70)	6	11.5	11.275	0.004	9	10.5	19.148	< 0.001	14	6.5	1.7988	0.407
	Undergraduate	564 (60.13)	8	7			12	6			14	4		
	Graduate or higher	283 (30.17)	7	9			11	7			14	6		
Age	< = 21	217 (23.13)	7	8	9.6578	0.008	11	7	12.946	0.002	14	4	2.8075	0.246
	22–26	551 (58.74)	8	8			12	7			14	4.5		
	> 26	170 (18.12)	7	8			10	6			15	5		
Division	Sylhet	366 (39.02)	7	6	16.83	0.010	11	6	9.2415	0.160	15	4	8.2736	0.219
	Rangpur	64 (6.82)	8	7			12	7			14	5		
	Rajshahi	53 (5.65)	9	7			12	5.75			13	6		
	Chattogram	137 (14.61)	8	8			11	7.5			14	4.5		
	Dhaka	189 (20.15)	6	7			11	8			14	5		
	Khulna	70 (7.46)	7	8.5			10	6.25			13	5		
	Mymensingh	59 (6.29)	8	10			12	7			15	5		

### 3.2 Reliability Analysis

Cronbach's *α* indicated good internal consistency for the knowledge and attitude scales, with values of 0.80 (95% CI: 0.78–0.81) and 0.87 (95% CI: 0.86–0.88), respectively. The willingness to participate in preventive practices scale had a Cronbach's *α* of 0.65 (95% CI: 0.60–0.69), which suggests acceptable but relatively lower reliability.

### 3.3 Knowledge of Participants About Mpox

**Figure 1:**
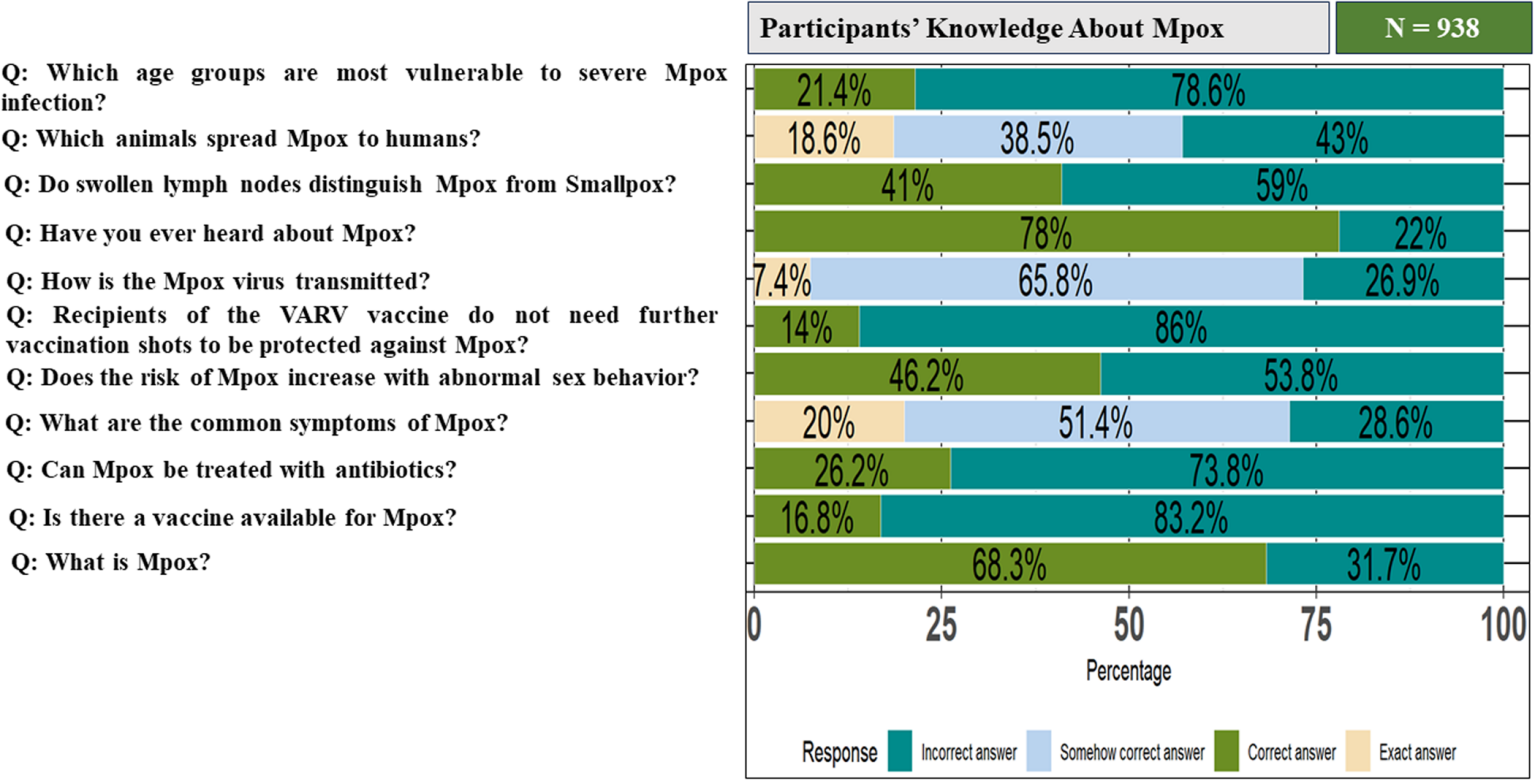
Showing participants' knowledge regarding Mpox: here “Exact answer” means the participants chose all the correct answers, “Correct answer” means the participants chose correct option, “Somehow correct answer” means the participants answered some of the options correctly, “Incorrect answer” means they did not answer any correct options/correct option. *Note:* VARV vaccine means smallpox (vaccinia virus) vaccine.

Among all the participants, only 7.25% (68/938) had good knowledge of Mpox, while 92.75% (870/938) demonstrated poor knowledge. In terms of knowledge‐related questions, more than 65% of the respondents were aware of two key questions: “Have you ever heard of Mpox?” and “What is Mpox?”. Approximately 50% of the participants were aware of the differential diagnosis between smallpox and Mpox, as well as the risks associated with sexual partners. However, knowledge was lower regarding vaccine availability (16.8%), treatment (26.2%), age vulnerability (21.4%), and the necessity of the Mpox vaccine (14%) (Figure 1).

The possible minimum and maximum achievable knowledge score ranged from 0 to 19 and the median knowledge score of the participants was 8 (IQR = 8). The Wilcoxon test found no statistically significant difference in knowledge scores based on gender (*p* value = 0.06) and area (*p* value = 0.12). However, the Kruskal–Wallis test revealed significant differences in knowledge scores based on education level (*p* value = 0.004), age group (*p* value = 0.008), and division (*p* value = 0.009), which is shown in Table 2.

### 3.4 Attitude of Participants Towards Mpox

**Figure 2:**
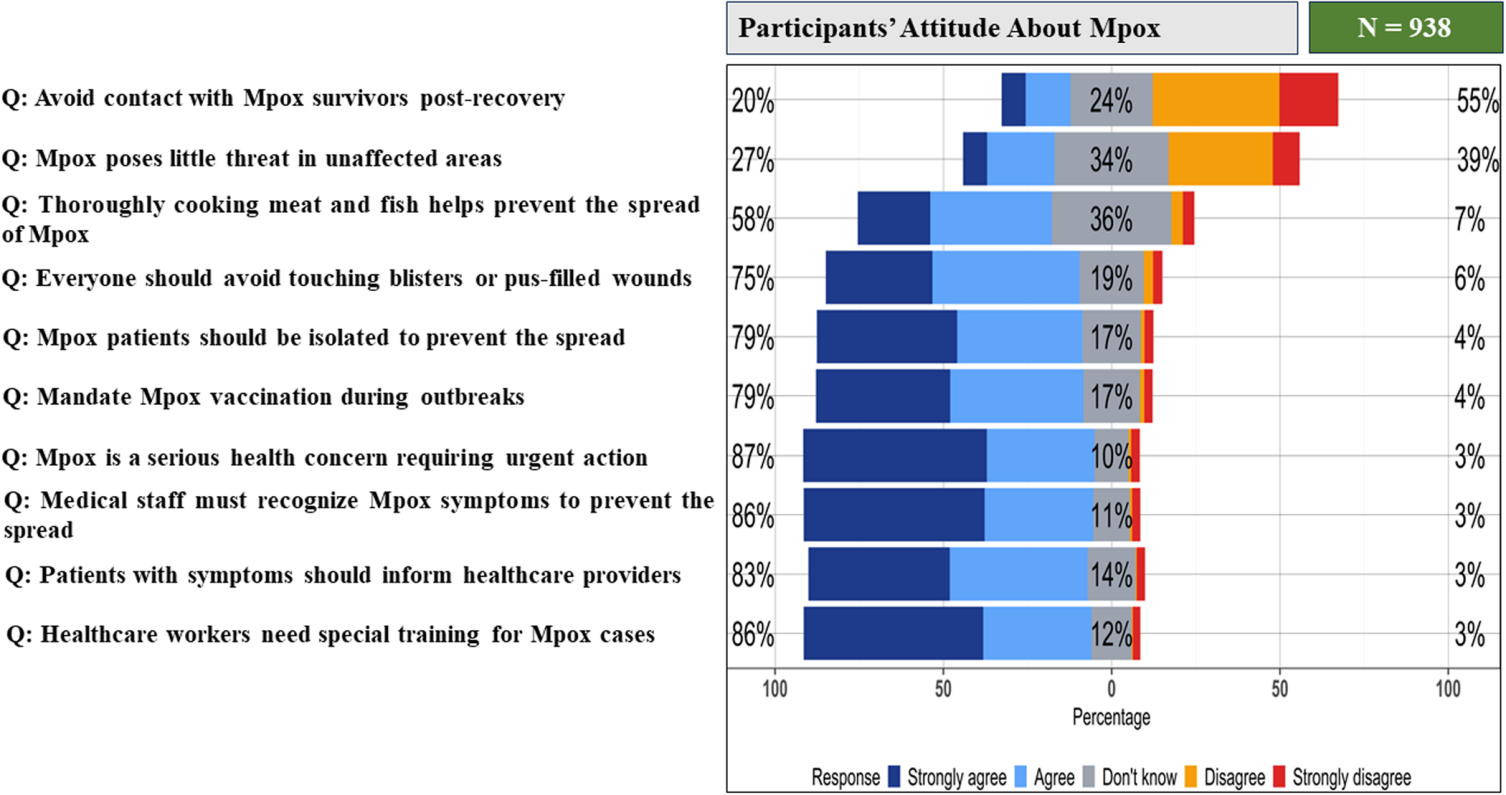
Showing the participants' attitudes regarding Mpox visualized by a 5‐point Likert plot answer ranging from “Strongly agree” to “Strongly disagree”.

A total of 80.6% of participants agreed that “Mpox is a serious health concern requiring urgent action”. Considerable support was also noted for the isolation of Mpox patients (79.4%), providing special training for healthcare workers (85.5%), identifying symptoms by them (86%), and informing them about symptoms by patients (82.9%). Additionally, 75.4% agreed that everyone should avoid touching blisters or wounds, and 78.9% supported mandating vaccination during outbreaks. Conversely, only 57.7% agreed that thoroughly cooking meat and fish helps prevent Mpox spread, 38.9% failed to agree that “Mpox poses little threat in unaffected areas,” and 55.11% were uncertain about avoiding contact with Mpox survivors (Figure 2).

The maximum achievable attitude score was 20, the minimum was 0, and participants' had a median attitude score of 12 (IQR = 7). There was no significant difference in attitude scores based on gender (*p* value = 0.90), and division (*p* value = 0.16) (Figure 4). However, the Kruskal–Wallis test revealed statistically significant differences in attitude scores based on area (*p* value = 0.003), education level (*p* value < 0.001), and age group (*p* value = 0.002), as shown in Table 2.

### 3.5 Practice of Participants Towards Monkeypox

**Figure 3:**
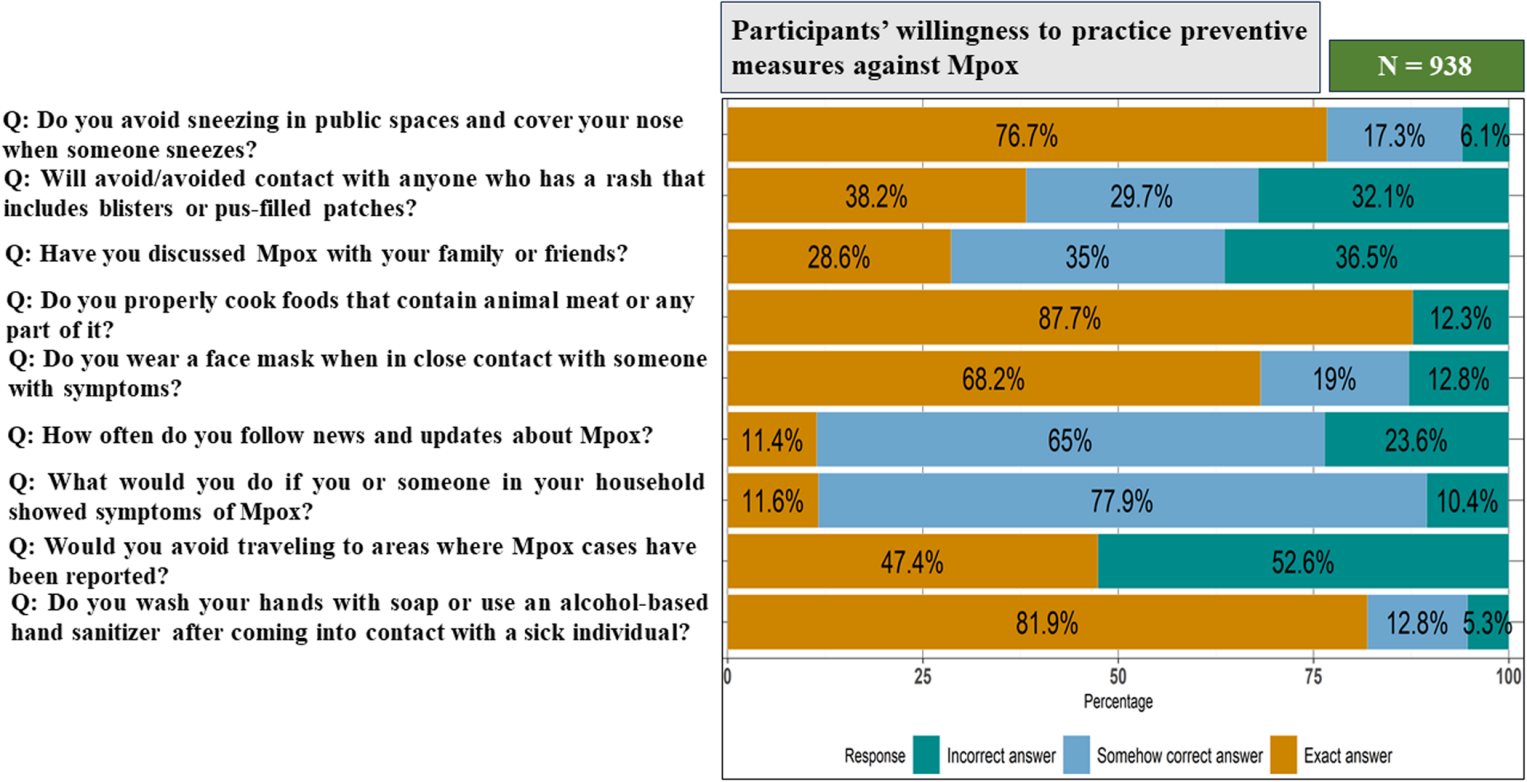
Showing the participants' knowledge regarding Mpox: here “Exact answer” means the participants chose the correct answer/all the correct answers, “Somehow correct answer” means the participants answered some of the options correctly, “Incorrect answer” means they did not answer any correct options/correct option.

Overall, 25.1% (235/938) of the participants demonstrated willingness to perform good preventive practice, while 75% (793/938) of the participants had unsatisfactory practice scores (Table 3). Our survey demonstrated gaps in Mpox prevention practices among participants. Notably, 32.1% did not avoid or had never avoided close contact with individuals exhibiting rashes, blisters, or pus‐filled patches. Additionally, 12.8% neglected wearing face masks when interacting with symptomatic individuals, and 5.3% did not wash their hands after contact with sick people. 10.4% were unaware of the appropriate steps to take when someone is affected by Mpox. Furthermore, 23.6% never sought updates on Mpox, while 36.5% had no intention of discussing it with their family. Notably, 6.1% did not practice proper sneezing hygiene in public places. Overall, participants' practice scores varied, but most failed to reach a satisfactory level. A majority of the participants (87.7%) reported consuming thoroughly cooked fish and meat, and 47.4% expressed concerns about traveling to areas with an Mpox outbreak (Figure 3).

**Table 3:** Knowledge, attitude, and preventive practice level of participants.

Level	Knowledge (*n*, %)	Attitude (*n*, %)	Practice (*n*, %)
Good/positive	68 (7.25)	193 (20.58)	235 (25.05)
Poor/negative	870 (92.75)	745 (79.42)	703 (74.95)

**Figure 4:**
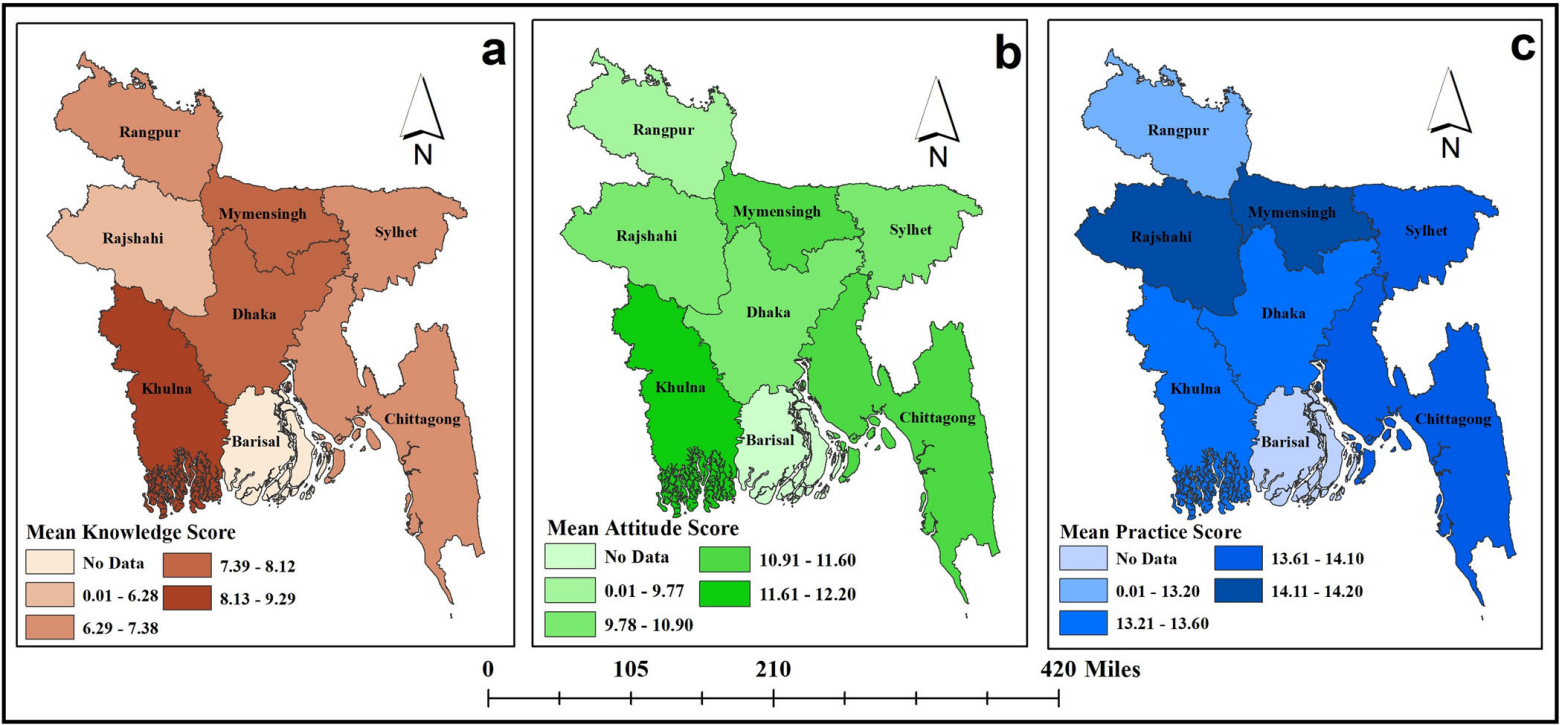
Distribution of (a) knowledge, (b) attitude, and (c) practice scores across different administrative divisions of Bangladesh as reported by the study participants.

In case of practice, the achievable lowest and highest scores ranged from 0 to 22, with a median practice score of 14 (IQR = 4.75) among participants. The Wilcoxon test reported statistically significant difference in practice scores based on gender (*p* value < 0.001), but did not find any significant difference based on area (*p* value = 0.37) and the Kruskal–Wallis test did not find any statistically significant difference in practice scores based on education level (*p* value = 0.40), age group (*p* value = 0.25), and division (*p* value = 0.22) (Table 2 and Figure 4).

### 3.6 Correlation Among Knowledge, Attitude, and Practice

The Spearman rank correlation revealed moderate positive correlations with strong statistical significance between each module's knowledge and attitude (*r*_s _= 0.53, *p* value < 0.001), knowledge and practice (*r*_s _= 0.41, *p* value < 0.001), and correlation between attitude and practice (*r*_s _= 0.40, *p* value < 0.001).

### 3.7 Univariate and Multivariable Logistic Regression

The univariate analysis revealed that gender, knowledge, and attitude scores were significantly associated (*p* value < 0.001) with the outcome variable level of willingness to practice. Females were significantly more likely to engage in good practices to prevent Mpox (OR = 1.8, 95% CI: 1.4–2.5; *p* value < 0.001). Participants with good knowledge had 5.3 times higher odds of engaging in practice preventive behavior (OR = 5.3, 95% CI: 3.2–8.8; *p* value < 0.001), while those with a positive attitude had 2.9 times higher odds (OR = 2.9, 95% CI: 2.1–4.1; *p* value < 0.001). Educational level, division, and urban residence were not significant predictors.

After adjusting for potential confounders, the multivariable model confirmed that gender, knowledge, and attitude scores remained statistically significant predictors of the outcome variable. Female participants continued to exhibit higher odds (aOR = 2.1, 95% CI: 1.6–2.9; *p* value < 0.001). Participants with good knowledge had 4.6 times higher odds of engaging in good preventive practice (aOR = 4.6, 95% CI: 2.7–7.8; *p* value < 0.001), while those with a positive attitude had 2.8 times higher odds of getting good preventive practice (aOR = 2.8, 95% CI: 2–4, *p* value < 0.001) (Table 4).

**Table 4:** Univariate and multivariable analysis of predictors of the level of preventive practice among participants.

Predictors		Odds ratio (95% CI)	*p* value	Adjusted odds ratio (95% CI)	*p* value
Gender	Male	1	< 0.001	1	< 0.001
	Female	1.84 (1.36–2.48)		2.14 (1.55–2.94)	
Area	Urban	1.12 (0.81–1.57)	0.490		
	Rural	1			
Education	Higher secondary or less	1.10 (0.67–1.83)	0.696		
	Undergraduate	1			
	Graduate or higher	1.07 (0.77–1.49)	0.674		
Age	> 26	1.51 (0.95–2.39)	0.082	1.96 (1.20–3.20)	0.007
	22–26	1.20 (0.83–1.76)	0.324	1.23 (0.83–1.84)	0.307
	< = 21	1		1	
Knowledge level	Good	5.29 (3.17–8.82)	< 0.001	4.58 (2.68–7.83)	< 0.001
	Poor	1		1	
Attitude level	Positive	2.94 (2.10–4.12)	< 0.001	2.82 (1.97–4.03)	< 0.001
	Negative	1		1	

Model diagnostics indicated no evidence of multicollinearity, as all VIF values were below 3. The Hosmer–Lemeshow goodness‐of‐fit test yielded a nonsignificant *p* value, confirming an adequate model fit. Examination of residual plots and Cook's distance revealed no concerning residuals or influential observations. The model demonstrated acceptable predictive ability, with an area under the ROC curve of 0.70.

## 4 Discussion

In recent years, Mpox has become a global public health concern due to the continuously increasing incidence and emergence in non‐endemic countries. As Bangladesh is one of the densely populated countries with frequent cross‐border movements with neighboring countries, especially with India, an outbreak of Mpox in Bangladesh will pose a serious risk to the less developed health care system. Although Mpox is a moderately contagious disease, the dense population of Bangladesh with limited knowledge, attitude, and willingness to practice preventive behavior can facilitate the transmission risk. This study can serve as a reference for the baseline information to better understand the KAP level of Mpox and to make informed decisions on adopting targeted health education and policy.

One of the key findings of this study was the identification of substantial gaps in knowledge and attitude against Mpox, with less than 21% of the participants demonstrating satisfactory knowledge and attitude level. Secondly, approximately 25% of the study population expressed an interest in maintaining good preventive practices, indicating low adoption of recommended health behavior. Lastly, results from multivariable logistic regression revealed that satisfactory Mpox preventive practice was significantly associated with gender, age, positive attitude, and proper knowledge. These findings highlight critical gaps in Mpox awareness among the people of Bangladesh, underscoring several areas that require attention. To the best of our knowledge, this is the first comprehensive assessment of willingness to adopt preventive practices towards Mpox in Bangladesh.

Given the public health threat posed by the emergence of Mpox, assessing the mass population's knowledge is of utmost importance, and to achieve this, the survey comprises 11 knowledge‐related questions. Median knowledge scores varied based on the education level and age of the participants. Overall, only 7.3% of the respondents showed satisfactory knowledge about Mpox in our study, which is lower than the previous findings conducted among Egyptian and Lebanese health professionals [19, 20]. This discrepancy may be due to the fact that the general public is less informed about emerging health‐related issues compared to students and healthcare workers [21]. Similarly, a comparable level of knowledge (6.3%) was reported from a study among Pakistani university students regarding Mpox transmission, prevention, and treatment. This might stem from similarity in awareness level, education level, the surrounding environment, and public health infrastructure [15]. In contrast, individuals in developed countries have better knowledge regarding Mpox, with 48.9% and 51.8% of knowledge‐related questions correctly answered by respondents from the USA [18] and Italy, respectively [22]. The lack of knowledge as reported in our study is concerning, as effective implementation of preventive measures to manage and treat potential outbreaks depends on public participation [23]. Moreover, identifying knowledge gaps is essential, but it is equally important to focus on those that are most relevant to the particular Mpox clade affecting a region to understand their transmission dynamics and to detect epidemiologically linked cases and outbreaks. For example, a study conducted in Saudi Arabia assessed phsicians knowledge and attitudes regarding a Mpox clade primarily associated with zoonotic transmission, however the questionnaire did not include items related to sexual transmission, which has emerged as a important and concerning route of Mpoxspread in recent outbreaks [24, 25]. These varying transmission pathways of different Mpox clades highlight the need for context‐specific knowledge to ensure effective prevention, diagnosis, and treatment [16].

Our study found that attitude scores varied based on respondents' area of residence, their education level, and age. In seven of the ten attitude‐related questions, at least 60% of participants expressed a positive attitude. However, efforts should be focused on improving attitudes towards avoiding contact with Mpox survivors' post‐recovery and recognizing the possible threats of Mpox in unaffected areas. Moreover, overall attitude level was not safisfactoy among current study participants which needs attention from policy makers. Only 20.4% of the respondents in our study had positive attitudes, which is lower than the studies conducted in Ethiopia [26], Pakistan [27], Lebanon [20], and Nepal [28]. These variations may be attributed to differences in cutoff values for assessing attitude levels, sample sizes, study design, demographic factors, and study settings.

Our study revealed that only a quarter of participants expressed interest in adopting proper preventive measures against Mpox. A study conducted in India reported higher and more satisfactory practice scores among participants compared to our findings [29]. The multivariable logistic regression identified gender, age, knowledge, and attitude level as significant predictors of preventive practices. Male participants were less likely to engage in good Mpox prevention practices, which is supported by another study among university students from Bangladesh, where female participants demonstrated better practice scores, despite having lower attitude scores [4]. Our study did not identify any statistically significant association (*p* value = 0.4) between satisfactory practice scores and education level. However, another study from Lebanon found that individuals with postgraduate degrees achieved more satisfactory results [20]. This prioritizes the people who should be focused on when making targeted interventions among risk groups for Mpox prevention and control during an outbreak.

Current research has identified several factors influencing Mpox‐related knowledge, attitudes, and practices, which are essential for developing effective prevention and control strategies. Moreover, approximately 46% of the participants were female, providing a balanced representation of both genders, and a substantial portion of the participants resided in rural areas. Though participants were from seven out of eight divisions, some divisions were not well represented, and the sample size was low in certain divisions. Furthermore, the study results might have overestimated the knowledge, attitude, and practice scores, as this study did not include any participants without formal education. Moreover, convenience sampling may lead to the overrepresentation of people interested in health‐related topics. Hence, the findings from this study cannot be generalized to the entire population, where a probability sampling, like stratified random sampling, would be a better model. Another limitation of our study is that we did not formally validate the questionnaire in the Bangladeshi general population. However, the questionnaire was carefully adapted from previously published studies, which we believe supports its relevance and content validity for this context. As the study relied on self‐reported data, social desirability bias might have also been an influencing factor. The participants might have overreported good practices and underreported risky behaviors to present themselves positively. Overall, the study revealed a substantial gap in knowledge, attitude, and practice (KAP) scores regarding Mpox among participants in Bangladesh. This underscores the urgent need for targeted awareness programs to secure a clear conception of people for better preparedness against Mpox in Bangladesh.

## 5 Conclusion

This study was aimed to determine the KAP levels regarding Mpox among people in Bangladesh, to identify potential risk factors, and to uncover the predictors associated with the outcome variable. The study revealed a substantial gap in knowledge, attitudes, and practices among the participants. Gender, knowledge scores, and attitude scores were significant predictors of the outcome variable, intention to practice. As the study results might be overestimated due to the overrepresentation of participants and social desirability bias, future studies should consider using stratified random sampling for more accurate results. However, the current study findings underscore the urgent need for immediate awareness programs in Bangladesh. This study can act as preliminary research, and the results can be used to inform policymakers for proper risk communication and educating the most vulnerable groups.

## Author Contributions

**Radwan Raquib:** conceptualization, data collection, data curation, formal analysis, methodology, resources, visualization, writing – original draft, writing – review and editing. **Proloy Chakraborty Tusher:** conceptualization, data collection, methodology, writing – review and editing. **Anisa Ahmed Chowdhury:** data curation, formal analysis, methodology, resources, visualization, writing – review and editing. **Obaidul Islam:** conceptualization, data collection, methodology, writing – review and editing, supervision. **Jahid Hasan Tipu:** conceptualization, methodology, resources, writing – review and editing, supervision. **Himel Talukder:** methodology, resources, writing – review and editing. **Mohammod Misbah Uddin:** methodology, writing – review and editing. **Ahsan Raquib:** conceptualization, methodology, data curation, formal analysis, resources, writing – review and editing, supervision. **Tilak Chandra Nath:** review and editing, supervision.

## Conflicts of Interest

The authors declare no conflicts of interest.

## Transparency Statement

The lead author Radwan Raquib, affirms that this manuscript is an honest, accurate, and transparent account of the study being reported; that no important aspects of the study have been omitted; and that any discrepancies from the study as planned (and, if relevant, registered) have been explained.

## Supporting information


**Supporting Table 1:** Questionnaire‐related Knowledge of Mpox. **Supporting Table 2:** Questionnaire‐related Attitude of participants regarding Mpox. **Supporting Table 3:** Questionnaire related to the willingness of preventive practice among participants.

## Data Availability

Data will be available upon reasonable request to the corresponding author.
