# Supplementary material for: Assessment of Knowledge, Attitude, and Practice Towards the Prevention of Monkeypox Among People of Bangladesh With at Least Primary Education: A Cross‐Sectional Study
Source: Health Sci Rep. 2025 Nov 26;8(12):e71560. doi: 10.1002/hsr2.71560 (PMC12657260; doi:10.1002/hsr2.71560)
Supplement: Supplementary file 1 — Supporting Table 1: Questionnaire‐related Knowledge of Mpox. Supporting Table 2: Questionnaire‐related Attitude of participants regarding Mpox. Supporting Table 3: Questionnaire related to the willingness of preventive practice among participants. [file HSR2-8-e71560-s001.docx]

**Supplementary Table 1:** Questionnaire-related Knowledge of Mpox.

| **Questions** | **Correct answer = 1** | **Incorrect answer = 0** |
| --- | --- | --- |
| Have you ever heard about Mpox? | Yes | Either No/Not sure |
| What is Mpox? | A viral disease | Either A bacterial infection/A parasitic infection/Don’t know |
| Is there a vaccine available for Mpox? | No | Either Yes/Don’t know |
| Can Mpox be treated with antibiotics? | No | Either Yes/Don’t know |
| Do swollen lymph nodes distinguish Mpox from Smallpox? | Yes | Either No/Don’t know |
| Recipients of the VARV vaccine do not need further vaccination shots to be protected against M. Pox | No | Either Yes/Don’t know |
| Does the risk of Mpox increase with abnormal sex behavior? | Yes | Either No/Don’t know) |
| Which age groups are most vulnerable to severe Monkeypox infection? | Children | Either Adults/Elderly/All age groups equally/Don’t know |
| CHECKBOX QUESTION:(Multiple answer) | | |
| How is Mpox virus transmitted? (Multiple answers possible) | [Close contact with infected animals, Human-to-human transmission through respiratory droplets, contact with contaminated surfaces or objects, Sexual contact] all mentioned routes are correct. Therefore, participants receive 1 mark for each correct response. Participants get a maximum of 4 marks by responding correctly and get 0 by responding “Don’t Know” | |
| What are the common symptoms of Mpox? (Multiple answers possible) | [Fever, Rash, Swollen lymph nodes, Headache, Muscle aches] all mentioned symptoms are correct. Therefore, participants receive 1 mark for each correct response. Participants get a maximum of 5 marks by responding correctly and get 0 by responding “Don’t Know”. | |
| Which animals spread Mpox to humans? (Multiple answers possible). | Correct answers: [Rodents (e.g., rats, squirrels), Primates (e.g., monkeys) ]. So, participants receive a maximum of 2 marks by answering them and 1 mark for each.  Incorrect answers: [Birds, Livestock (e.g., cattle, pigs), Don’t know]. So, participants receive 0 marks for answering them. | |

**Supplementary Table 2:** Questionnaire-related Attitude of participants regarding Mpox.

| **Question** | **Answer** |
| --- | --- |
| Mpox is a serious public health concern that requires urgent action. | The question revealed participants' attitudes regarding Mpox. Answers ranging from “Strongly agree” to “Strongly disagree”.  Mark: [Strongly agree = 2, Agree = 1, Don't Know/Disagree/Strongly Disagree = 0] |
| Thoroughly cooking meat and fish helps prevent the spread of Mpox. | The question revealed participants' attitudes regarding Mpox. Answers ranging from “Strongly agree” to “Strongly disagree”.  Mark: [Strongly agree = 2, Agree = 1, Don't Know/Disagree/Strongly Disagree = 0] |
| Mpox patients should be isolated to prevent the spread. | The question revealed participants' attitudes regarding Mpox. Answers ranging from “Strongly agree” to “Strongly disagree”.  Mark: [Strongly agree = 2, Agree = 1, Don't Know/Disagree/Strongly Disagree = 0] |
| Healthcare workers need special training for Mpox cases . | The question revealed participants' attitudes regarding Mpox. Answers ranging from “Strongly agree” to “Strongly disagree”.  Mark: [Strongly agree = 2, Agree = 1, Don't Know/Disagree/Strongly Disagree = 0] |
| Patients with symptoms should inform healthcare providers. | The question revealed participants' attitudes regarding Mpox. Answers ranging from “Strongly agree” to “Strongly disagree”.  Mark: [Strongly agree = 2, Agree = 1, Don't Know/Disagree/Strongly Disagree = 0] |
| Medical staff must recognize Mpox symptoms to prevent the spread. | The question revealed participants' attitudes regarding Mpox. Answers ranging from “Strongly agree” to “Strongly disagree”.  Mark: [Strongly agree = 2, Agree = 1, Don't Know/Disagree/Strongly Disagree = 0] |
| Avoid contact with Mpox survivors post-recovery? | The question revealed participants' attitudes regarding Mpox. Answers ranging from “Strongly agree” to “Strongly disagree”.  Mark: [Strongly disagree = 2, Disagree = 1, Don't Know/Agree/Strongly agree = 0] |
| Mpox poses little threat in unaffected areas. | The question revealed participants' attitudes regarding Mpox. Answers ranging from “Strongly agree” to “Strongly disagree”.  Mark: [Strongly disagree = 2, Disagree = 1, Don't Know/Agree/Strongly agree = 0] |
| Everyone should avoid touching blisters or pus-filled wounds. | The question revealed participants' attitudes regarding Mpox. Answers ranging from “Strongly agree” to “Strongly disagree”.  Mark: [Strongly agree = 2, Agree = 1, Don't Know/Disagree/Strongly Disagree = 0] |
| Mandate Mpox vaccination during outbreaks. | The question revealed participants' attitudes regarding Mpox. Answers ranging from “Strongly agree” to “Strongly disagree”.  Mark: [Strongly agree = 2, Agree = 1, Don't Know/Disagree/Strongly Disagree = 0] |

**Supplementary Table 3:** Questionnaire related to the willingness of preventive practice among participants.

| **Question** | **Answer** |
| --- | --- |
| Will you avoid/avoided close contact with anyone who has a rash that includes blisters or pus-filled patches? | Marks: [Yes = 3, Most often = 2, Sometimes = 1, No = 0] |
| Do you wash your hands with soap or use an alcohol-based hand sanitizer after coming into contact with a sick individual? | Marks: [Yes = 3, Most often = 2, Sometimes = 1, No = 0] |
| Do you wear a face mask when in close contact with someone with symptoms? | Marks: [Yes = 3, Most often = 2, Sometimes = 1, No = 0] |
| How often do you follow news and updates about Mpox? | Marks: [Daily = 3, Weekly = 2, Rarely = 1, Never = 0] |
| Would you avoid traveling to areas where Mpox cases have been reported? | Marks: [Yes = 1, No/Not sure = 0] |
| Have you discussed Mpox with your family or friends? | Marks: [Yes = 2, Not yet, but plan to = 1, No/No intention to discuss = 0] |
| Do you avoid sneezing in public spaces and cover your nose when someone sneezes? | Marks: [Yes = 3, Most often = 2, Sometimes = 1, No = 0] |
| Do you eat thoroughly cooked foods that contain animal meat or any part of it? | Marks: [Yes = 1, No/Not aware of the need to purchase = 0] |
| CHECKBOX (Multiple answers possible) | |
| What would you do if you or someone in your household showed symptoms of Monkeypox? (Multiple answers possible). | [Seek medical help immediately, Isolate the person, Use home remedies] all mentioned are correct. Therefore, participants receive 1 mark for each correct response. Participants get a maximum of 3 marks by responding correctly, and get 0 by responding [Don’t Know/Wait to see if symptoms worsen] |
